# Early detection of the emerging SARS-CoV-2 BA.2.86 lineage through integrated genomic surveillance of wastewater and COVID-19 cases in Sweden, weeks 31 to 38 2023

**DOI:** 10.2807/1560-7917.ES.2023.28.46.2300595

**Published:** 2023-11-16

**Authors:** Carmen Espinosa-Gongora, Carlo Berg, Moa Rehn, Javier Edo Varg, Lena Dillner, Neus Latorre-Margalef, Anna J Székely, Emmi Andersson, Elin Movert

**Affiliations:** 1 Public Health Agency of Sweden (Folkhälsomyndigheten), Solna, Sweden; 2 ECDC Fellowship Programme, Public Health Microbiology path (EUPHEM), European Centre for Disease Prevention and Control (ECDC), Stockholm, Sweden; 3 Swedish Environmental Epidemiology Center (SEEC), Uppsala, Sweden

**Keywords:** SARS-CoV-2, BA.2.86, whole genome sequencing, wastewater surveillance, Sweden, Freyja

## Abstract

The SARS-CoV-2 BA.2.86 Omicron subvariant was first detected in wastewater in Sweden in week 31 2023, using 21 highly specific markers from the 50 investigated. We report BA.2.86’s introduction and subsequent spread to all 14 regions performing wastewater sampling, and on 70 confirmed COVID-19 cases, along with the emergence of sublineages JN.1 and JN.2. Further, we investigated two novel mutations defining the unique BA.2.86 branching in Sweden. Our integrated approach enabled variant tracking, offering evidence for well-informed public health interventions.

In August 2023, the novel severe acute respiratory syndrome coronavirus 2 (SARS-CoV-2) BA.2.86 Omicron sublineage was first identified by public health surveillance programs in several European countries, Israel, South Africa and the United States [1-3]. The substantial genetic changes exhibited by BA.2.86, along with evidence of community transmission and international spread, led to its classification as a variant under monitoring (VUM) by the World Health Organization (WHO) [4] and the European Centre for Disease Prevention and Control (ECDC) thereafter [5]. Here, we present the results of an integrated genomic surveillance approach in Sweden, involving the identification of BA.2.86-specific markers in wastewater alongside the monitoring of COVID-19 cases with BA.2.86 from week 31 to week 38 in 2023.

## BA.2.86 marker specificity and wastewater analysis

We investigated the occurrence of BA.2.86-specific markers [6,7] across SARS-CoV-2 variants within the global GISAID-EpiCoV database [8] (see Supplementary methods for BA.2.86 marker specificity investigation), a variation of the VaQuERo sensitive marker approach [9]. Of 50 investigated markers, 21 were mostly (> 90%) reported in BA.2.86 genomes uploaded to GISAID since July 2023 (search results are included in Supplementary Figure S1).

**Figure 1:**
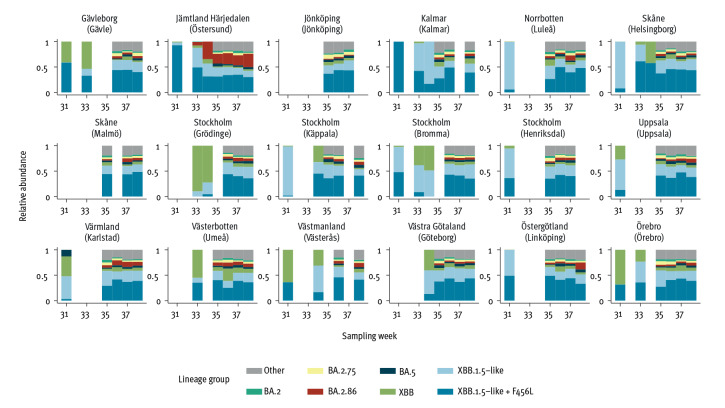
Relative abundance of SARS-CoV-2 BA.2.86 and other circulating variant groups in wastewater samples collected at 18 wastewater treatment plants in 14 of 21 regions, Sweden, weeks 31–38 2023 (n = 91 samples)

In Sweden, wastewater samples are collected weekly from 18 wastewater treatment plants (WWTP) distributed across 14 of 21 regions [10]. Sequenced wastewater samples with sufficient coverage (n = 91 from weeks 31–38) were analysed for the presence of the 21 highly specific BA.2.86 markers. Simultaneously, the Freyja algorithm [11] (cutoff and details described in the Supplementary methods) was employed to assign SARS-CoV-2 variants. In samples which were not assigned by Freyja, identification of at least one of the highly specific markers signified additional positive detections in wastewater, including the earliest detections in Östersund (week 31) and Örebro (week 33). Supplementary Figure S2 illustrates the presence of each BA.2.86 marker in all positive wastewater samples, including Freyja results. The relative abundance of BA.2.86 and other lineages assigned by Freyja is shown in Figure 1.

SARS-CoV-2: severe acute respiratory syndrome coronavirus 2.

The lineage groups, as assigned by the Freyja algorithm, are shown for each region. The cities where the wastewater treatment plants are located within each region are shown in parentheses.

## Analysis of samples from COVID-19 cases

**Figure 2:**
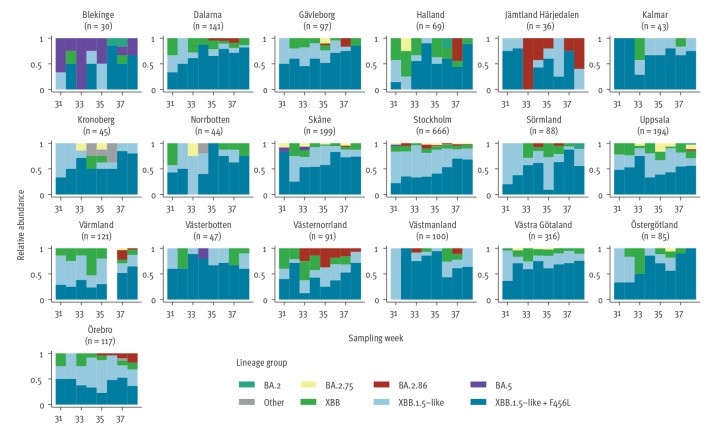
Relative abundance of SARS-CoV-2 BA.2.86 and other circulating variant groups in samples collected from COVID-19 cases in 19 of 21 regions, Sweden, weeks 31–38 2023 (n = 2,529 samples)

During the study period (weeks 31–38), 3,983 COVID-19 cases were detected in Sweden. At the data collection endpoint on 17 October 2023, 2,529 (63%) cases were sequenced and assigned a Phylogenetic Assignment of Named Global Outbreak (Pango) lineage. Characteristics of cases, vaccination status, hospitalisation and death data were obtained from national registries included in the routine surveillance of COVID-19, i.e. National Electronic Notification System for Notifiable Diseases - SmiNet (age and sex), National Vaccination Registry (vaccination status), National Patient Register (hospitalisation in ward), Swedish Intensive Care Registry (ICU), and Swedish Tax Agency (date of death). Distribution of age, sex, hospitalisation, 30-day mortality and vaccination status did not differ between cases infected with BA.2.86 (n = 70) versus other SARS-CoV-2 lineages where registry metadata were available (n = 2,171) during the same period. A description of BA.2.86 cases can be found in Supplementary Table S1. The weekly distribution of lineages among samples from COVID-19 cases is presented by region in Figure 2.

SARS-CoV-2: severe acute respiratory syndrome coronavirus 2.

The total number (n) of samples from individuals analysed in weeks 31–38 is indicated for each region.

The first cases with BA.2.86 infections in Sweden were sampled in Region Stockholm in week 32 and were the earliest samples on a global scale that belonged to the BA.2.86.1 novel branches carrying amino acid substitutions S:T299I or ORF1a:Y621C [12,13]. The mutation S:T299I in BA.2.86.1, exclusive to Sweden, formed a phylogenetic cluster with 16 of 70 BA.2.86 cases (data not shown). The mutation ORF1a:Y621C, designated as lineage JN.2 on 12 October 2023 [14], formed a cluster with 50 of 70 BA.2.86 cases in Sweden, and can also be found in other European countries, Asia and North America [12]. Lineage JN.1, characterised in part by mutation S:L455S, had not been detected in any cases in Sweden up to sampling week 38. Freyja analysis indicated low frequency circulation of JN.1 (1–4%) at most WWTPs during weeks 35–38 (Supplementary Figure S2). As convergent amino acid changes at position L455 have been observed in contemporary XBB.1.5-like and BA.2.75 descendant lineages [5] and have been linked to reduced binding of neutralising antibodies [15], we will continue monitoring the emergence of JN.1 or the possible introduction of S:L455S in other BA.2.86 sublineages.

## Detection and timeline of BA.2.86 spread 

Based on the analysis of BA.2.86-specific markers, the first signal for BA.2.86 was detected in wastewater from the Östersund WWTP in Region Jämtland Härjedalen, in week 31. The first two cases infected with BA.2.86 were detected in Region Stockholm in week 32, followed by cases in four additional regions, including Jämtland Härjedalen, over the next 2 weeks. By week 36, seven regions had detected cases, and all 14 regions with sampled WWTPs had positive BA.2.86 signals in wastewater. At the data collection endpoint, BA.2.86 had been detected in 70 COVID-19 cases tested across 12 regions, representing 2.8% of all cases with available sequence results.

**Figure 3:**
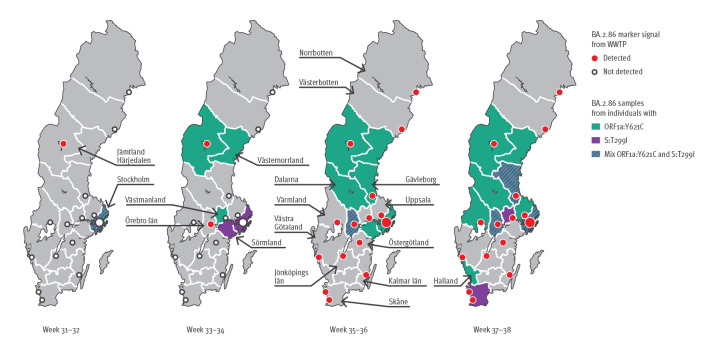
BA.2.86 spread in samples from wastewater treatment plants and COVID-19 cases, Sweden, weeks 31–38 2023

SARS-CoV-2: severe acute respiratory syndrome coronavirus 2; WWTP: wastewater treatment plants.

The two novel BA.2.86 sublineage mutations, ORF1a:Y621C and S:T299I, identified in samples from COVID-19 cases were also detected in wastewater. Specifically, ORF1a:Y621C was detected at the WWTP in Östersund for 6 consecutive weeks (33–38), and at all sampled WWTPs starting from week 35. The S:T299I mutation was only detected sporadically at different WWTPs between weeks 35–38 and for the longest consecutive period in Gävle during weeks 36–38. Figure 3 illustrates the detection of BA.2.86 in samples from wastewater (presence of BA.2.86-specific markers) and COVID-19 cases over time, including geographical spread and presence of sublineage-specific mutations.

Region names are indicated upon the initial BA.2.86 detection. The larger red dot in Region Stockholm represents four WWTPs.

## Discussion

The current SARS-CoV-2 testing and sequencing strategy in Sweden targets those at risk of severe disease. With addition of genomic wastewater surveillance, early warning systems can be expanded, and community-level monitoring of emerging variants is enabled. The Freyja tool, used in our investigations, is a robust method ideal for reporting SARS-CoV-2 circulating variants in wastewater. However, its algorithm requires the fulfilment of certain criteria for lineage designation, including half of the mutations differentiating that lineage from its closest relative, among sites covered by genome sequencing [11], and relies on official lineage designation updates. The detection of BA.2.86-associated mutations in wastewater [2,9] in combination with our marker specificity assessment enhanced sensitivity, which resulted in our first detection in Sweden and provided an internal early warning for the presence of BA.2.86. Furthermore, this approach allowed us to track the emergence of the novel branch carrying amino acid substitution ORF1a:Y621C before it was designated JN.2. Implementation of this highly sensitive approach relies greatly on the comprehensive collection of global SARS-CoV-2 genomes of novel variants in humans.

Our findings agree with the observed rapid evolution of BA.2.86 [1], displaying newly acquired mutations among samples from COVID-19 cases, including amino acid substitutions exclusive to Sweden. Additionally, our data suggest a steady introduction of BA.2.86 into the country, with notable viral circulation before the reporting of cases, and with comparable disease outcomes to other concurrent lineages.

## Conclusion

Sequencing of wastewater and assessment of marker specificity can be used as a surveillance tool for early detection and monitoring of community spread of emerging SARS-CoV-2 variants. By integrating surveillance and research data on virulence and immune evasion characteristics of emerging variants, we could generate robust evidence to guide public health actions. However, in-depth genomic wastewater surveillance for public health action as proposed in this report relies on the global sharing of high-quality genomes of emerging SARS-CoV-2 variants.

## Supplementary Data

1199000 Supplement
