# Supplementary material for: Early detection of the emerging SARS-CoV-2 BA.2.86 lineage through integrated genomic surveillance of wastewater and COVID-19 cases in Sweden, weeks 31 to 38 2023
Source: Euro Surveill. 2023 Nov 16;28(46):2300595. doi: 10.2807/1560-7917.ES.2023.28.46.2300595 (PMC10655203; doi:10.2807/1560-7917.ES.2023.28.46.2300595)
Supplement: Supplement [file 23-00595_MOVERT_Supplement.pdf]

This supplementary material is hosted by Eurosurveillance as supporting information alongside the article 'Early detection of the emerging BA.2.86 lineage in Sweden through integrated genomic surveillance of wastewater and SARS-CoV-2 cases', on behalf of the authors, who remain responsible for the accuracy and appropriateness of the content. The same standards for ethics, copyright, attributions and permissions as for the article apply. Supplements are not edited by Eurosurveillance and the journal is not responsible for the maintenance of any links or email addresses provided therein.

Supplementary figures and tables

Supplementary Figure S1. Percentage of genomes in the GISAID database (last accessed 2023-10-19) containing each of the BA.2.86 branch-specific markers, belonging to BA.2.86 lineages compared to other circulating groups of SARS-CoV-2 lineages.

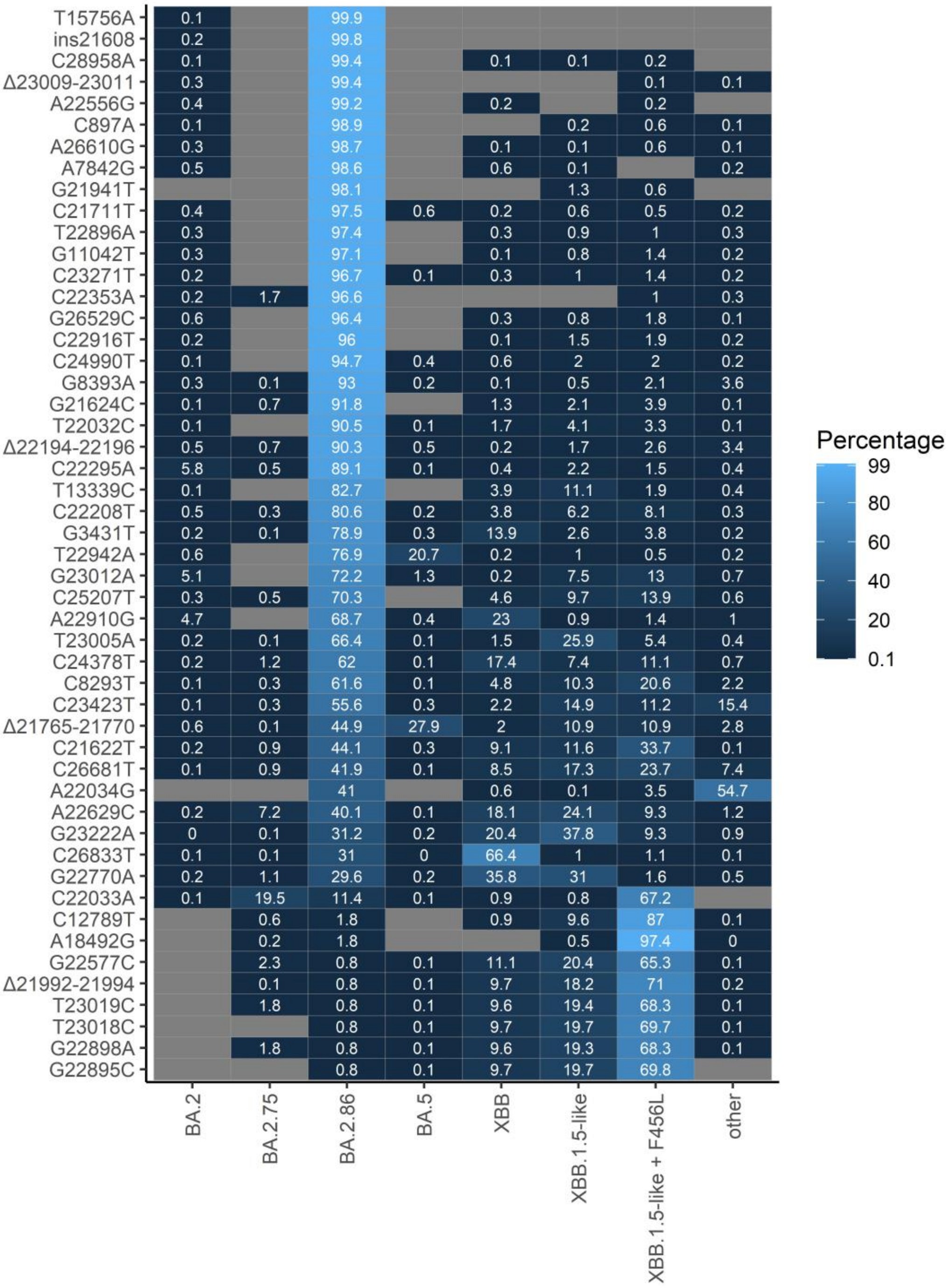

Supplementary Figure S2. Relative abundance of BA.2.86 lineages and of each BA.2.86-specific marker in wastewater samples collected from 18 WWTPs in Sweden. Markers were detected using iVar and the presence and abundance of BA.2.86 lineages were estimated by the Freyja algorithm (coverage cutoff 40).

This supplementary material is hosted by Eurosurveillance as supporting information alongside the article 'Early detection of the emerging BA.2.86 lineage in Sweden through integrated genomic surveillance of wastewater and SARS-CoV-2 cases', on behalf of the authors, who remain responsible for the accuracy and appropriateness of the content. The same standards for ethics, copyright, attributions and permissions as for the article apply. Supplements are not edited by Eurosurveillance and the journal is not responsible for the maintenance of any links or email addresses provided therein.

	Freyja		Relative abundance of BA.2.86-specific markers																				
week	lineage	abundance	T15756A	ins21608	C28958A	Δ23009-23011	A22556G	C897A	A26610G	A7842G	G21941T	C21711T	T22896A	G11042T	C23271T	C22353A	G26529C	C22916T	C24990T	G8393A	G21624C	T22032C	Δ2ss2194-22196
Östersund																							
38	JN.2	0.24	0.25	0.36	0.29	0.12	0.37	0.31	0.34	0.30	0.26	0.50	0.27	0.31	0.32	0.23	0.23	0.33	0.16	0.37	0.37	0.27	0.32
38	JN.1	0.02	0.25	0.36	0.29	0.12	0.37	0.31	0.34	0.30	0.26	0.50	0.27	0.31	0.32	0.23	0.23	0.33	0.16	0.37	0.37	0.27	0.32
37	JN.2	0.12	0.21	0.05	0.17	0.05	0.27	0.24	0.12	0.21	0.21	0.38	0.24	0.22	0.23	0.15	0.10	0.28	0.10	0.28	0.05	0.18	0.07
37	JN.3	0.06	0.21	0.05	0.17	0.05	0.27	0.24	0.12	0.21	0.21	0.38	0.24	0.22	0.23	0.15	0.10	0.28	0.10	0.28	0.05	0.18	0.07
37	JN.1	0.01	0.21	0.05	0.17	0.05	0.27	0.24	0.12	0.21	0.21	0.38	0.24	0.22	0.23	0.15	0.10	0.28	0.10	0.28	0.05	0.18	0.07
36	JN.2	0.20	0.25	0.14	0.24	0.10	0.51	0.24	0.32	0.18	0.20	0.47	0.22	0.28	0.23	0.23	0.23	0.29	0.14	0.27	0.16	0.22	0.27
36	JN.1	0.02	0.25	0.14	0.24	0.10	0.51	0.24	0.32	0.18	0.20	0.47	0.22	0.28	0.23	0.23	0.23	0.29	0.14	0.27	0.16	0.22	0.27
35	JN.2	0.16	0.32	0.10	0.21	0.07	0.44	0.19	0.21	0.18	0.22	0.53	0.19	0.24	0.25	0.13	0.20	0.24	0.11	0.23	0.12	0.21	0.17
35	JN.1	0.03	0.32	0.10	0.21	0.07	0.44	0.19	0.21	0.18	0.22	0.53	0.19	0.24	0.25	0.13	0.20	0.24	0.11	0.23	0.12	0.21	0.17
34	JN.2	0.35	NA	NA	0.13	0.45	NA	1.00	NA	NA	0.44	0.87	NA	0.80	0.22	1.00	NA	NA	NA	0.35	NA	0.37	0.63
33	JN.2	0.07	NA	0.02	0.24	0.12	NA	NA	NA	0.22	0.07	NA	NA	0.27	0.06	1.00	NA	NA	NA	NA	0.01	0.05	0.50
31	NA	NA	NA	NA	NA	NA	NA	NA	NA	0.53	NA	NA	NA	NA	0.08	NA	NA	NA	NA	NA	NA	NA	NA
Örebro																							
38	JN.2	0.03	0.15	NA	0.08	NA	0.07	0.07	NA	0.06	0.05	0.22	0.09	0.07	0.03	0.07	0.05	0.12	0.07	0.06	0.08	0.17	0.04
38	JN.1	0.02	0.15	NA	0.08	NA	0.07	0.07	NA	0.06	0.05	0.22	0.09	0.07	0.03	0.07	0.05	0.12	0.07	0.06	0.08	0.17	0.04
38	BA.2.86.3	0.00	0.15	NA	0.08	NA	0.07	0.07	NA	0.06	0.05	0.22	0.09	0.07	0.03	0.07	0.05	0.12	0.07	0.06	0.08	0.17	0.04
37	JN.1	0.02	0.08	NA	0.04	NA	0.03	0.05	NA	0.04	0.03	NA	0.09	0.08	0.02	0.04	0.04	0.13	0.07	0.06	NA	0.15	NA

This supplementary material is hosted by Eurosurveillance as supporting information alongside the article 'Early detection of the emerging BA.2.86 lineage in Sweden through integrated genomic surveillance of wastewater and SARS-CoV-2 cases', on behalf of the authors, who remain responsible for the accuracy and appropriateness of the content. The same standards for ethics, copyright, attributions and permissions as for the article apply. Supplements are not edited by Eurosurveillance and the journal is not responsible for the maintenance of any links or email addresses provided therein.

	Freyja		Relative abundance of BA.2.86-specific markers																				
week	lineage	abundance	T15756A	ins21608	C28958A	Δ23009-23011	A22556G	C897A	A26610G	A7842G	G21941T	C21711T	T22896A	G11042T	C23271T	C22353A	G26529C	C22916T	C24990T	G8393A	G21624C	T22032C	Δ2ss2194-22196
37	JN.2	0.01	0.08	NA	0.04	NA	0.03	0.05	NA	0.04	0.03	NA	0.09	0.08	0.02	0.04	0.04	0.13	0.07	0.06	NA	0.15	NA
37	BA.2.86.3	0.00	0.08	NA	0.04	NA	0.03	0.05	NA	0.04	0.03	NA	0.09	0.08	0.02	0.04	0.04	0.13	0.07	0.06	NA	0.15	NA
36	JN.2	0.02	NA	NA	0.09	NA	NA	NA	NA	0.08	0.03	NA	0.08	0.07	NA	0.08	0.09	0.12	0.05	0.12	NA	0.14	NA
36	JN.1	0.01	NA	NA	0.09	NA	NA	NA	NA	0.08	0.03	NA	0.08	0.07	NA	0.08	0.09	0.12	0.05	0.12	NA	0.14	NA
36	BA.2.86.3	0.00	NA	NA	0.09	NA	NA	NA	NA	0.08	0.03	NA	0.08	0.07	NA	0.08	0.09	0.12	0.05	0.12	NA	0.14	NA
35	JN.1	0.02	0.07	NA	0.05	NA	NA	0.05	NA	0.05	0.03	0.10	0.09	0.03	NA	0.03	0.07	0.13	0.06	0.08	NA	0.15	NA
35	JN.2	0.01	0.07	NA	0.05	NA	NA	0.05	NA	0.05	0.03	0.10	0.09	0.03	NA	0.03	0.07	0.13	0.06	0.08	NA	0.15	NA
33	NA	NA	NA	NA	NA	NA	NA	NA	NA	NA	NA	NA	NA	0.07	NA	NA	NA	NA	NA	NA	NA	NA	NA
Västerås																							
38	JN.2	0.05	0.21	NA	0.11	0.02	0.07	0.08	0.06	0.09	0.10	0.24	0.11	0.18	0.09	0.06	0.14	0.13	0.09	0.19	NA	0.20	0.10
38	JN.1	0.03	0.21	NA	0.11	0.02	0.07	0.08	0.06	0.09	0.10	0.24	0.11	0.18	0.09	0.06	0.14	0.13	0.09	0.19	NA	0.20	0.10
38	BA.2.86.3	0.00	0.21	NA	0.11	0.02	0.07	0.08	0.06	0.09	0.10	0.24	0.11	0.18	0.09	0.06	0.14	0.13	0.09	0.19	NA	0.20	0.10
36	BA.2.86.3	0.02	0.09	NA	0.08	0.02	NA	0.04	0.08	0.06	0.06	NA	0.10	0.04	0.07	0.09	0.09	0.14	0.05	0.07	NA	0.17	0.09
36	BA.2.86	0.02	0.09	NA	0.08	0.02	NA	0.04	0.08	0.06	0.06	NA	0.10	0.04	0.07	0.09	0.09	0.14	0.05	0.07	NA	0.17	0.09
36	JN.1	0.02	0.09	NA	0.08	0.02	NA	0.04	0.08	0.06	0.06	NA	0.10	0.04	0.07	0.09	0.09	0.14	0.05	0.07	NA	0.17	0.09
36	JN.2	0.01	0.09	NA	0.08	0.02	NA	0.04	0.08	0.06	0.06	NA	0.10	0.04	0.07	0.09	0.09	0.14	0.05	0.07	NA	0.17	0.09
Uppsala																							
38	JN.2	0.05	0.17	0.21	0.08	0.01	0.09	0.10	0.04	0.07	0.09	0.27	0.13	0.10	0.08	0.09	0.09	0.16	0.07	0.09	0.22	0.15	0.04

This supplementary material is hosted by Eurosurveillance as supporting information alongside the article 'Early detection of the emerging BA.2.86 lineage in Sweden through integrated genomic surveillance of wastewater and SARS-CoV-2 cases', on behalf of the authors, who remain responsible for the accuracy and appropriateness of the content. The same standards for ethics, copyright, attributions and permissions as for the article apply. Supplements are not edited by Eurosurveillance and the journal is not responsible for the maintenance of any links or email addresses provided therein.

	Freyja		Relative abundance of BA.2.86-specific markers																				
week	lineage	abundance	T15756A	ins21608	C28958A	Δ23009-23011	A22556G	C897A	A26610G	A7842G	G21941T	C21711T	T22896A	G11042T	C23271T	C22353A	G26529C	C22916T	C24990T	G8393A	G21624C	T22032C	Δ2ss2194-22196
38	JN.1	0.02	0.17	0.21	0.08	0.01	0.09	0.10	0.04	0.07	0.09	0.27	0.13	0.10	0.08	0.09	0.09	0.16	0.07	0.09	0.22	0.15	0.04
38	BA.2.86	0.01	0.17	0.21	0.08	0.01	0.09	0.10	0.04	0.07	0.09	0.27	0.13	0.10	0.08	0.09	0.09	0.16	0.07	0.09	0.22	0.15	0.04
38	BA.2.86.3	0.00	0.17	0.21	0.08	0.01	0.09	0.10	0.04	0.07	0.09	0.27	0.13	0.10	0.08	0.09	0.09	0.16	0.07	0.09	0.22	0.15	0.04
37	JN.2	0.03	0.08	NA	0.06	NA	0.03	0.05	0.05	0.06	0.06	0.12	0.10	0.09	0.06	0.04	0.12	0.13	0.06	0.07	NA	0.15	NA
37	JN.1	0.02	0.08	NA	0.06	NA	0.03	0.05	0.05	0.06	0.06	0.12	0.10	0.09	0.06	0.04	0.12	0.13	0.06	0.07	NA	0.15	NA
37	BA.2.86.3	0.00	0.08	NA	0.06	NA	0.03	0.05	0.05	0.06	0.06	0.12	0.10	0.09	0.06	0.04	0.12	0.13	0.06	0.07	NA	0.15	NA
37	BA.2.86.2	0.00	0.08	NA	0.06	NA	0.03	0.05	0.05	0.06	0.06	0.12	0.10	0.09	0.06	0.04	0.12	0.13	0.06	0.07	NA	0.15	NA
36	JN.1	0.03	0.17	NA	0.05	NA	NA	0.06	NA	0.05	0.03	NA	0.09	0.07	NA	0.03	0.05	0.14	0.07	0.06	NA	0.12	NA
36	JN.2	0.00	0.17	NA	0.05	NA	NA	0.06	NA	0.05	0.03	NA	0.09	0.07	NA	0.03	0.05	0.14	0.07	0.06	NA	0.12	NA
35	JN.1	0.01	0.13	NA	0.05	NA	NA	0.08	NA	0.05	0.04	NA	0.07	0.03	NA	NA	0.04	0.10	0.03	0.08	NA	0.14	NA
35	BA.2.86.3	0.00	0.13	NA	0.05	NA	NA	0.08	NA	0.05	0.04	NA	0.07	0.03	NA	NA	0.04	0.10	0.03	0.08	NA	0.14	NA
31	JN.2	0.00	NA	NA	NA	NA	NA	NA	NA	NA	NA	NA	NA	NA	NA	NA	NA	NA	NA	NA	NA	NA	NA
Umeå																							
38	JN.2	0.05	0.20	0.06	0.09	0.01	0.09	0.09	0.04	0.09	0.11	0.39	0.09	0.13	0.05	0.06	0.07	0.13	0.08	0.11	0.09	0.17	0.04
38	JN.1	0.01	0.20	0.06	0.09	0.01	0.09	0.09	0.04	0.09	0.11	0.39	0.09	0.13	0.05	0.06	0.07	0.13	0.08	0.11	0.09	0.17	0.04
38	BA.2.86.3	0.00	0.20	0.06	0.09	0.01	0.09	0.09	0.04	0.09	0.11	0.39	0.09	0.13	0.05	0.06	0.07	0.13	0.08	0.11	0.09	0.17	0.04
37	JN.2	0.05	0.12	0.05	0.08	0.02	NA	0.13	0.04	0.08	0.07	0.29	0.11	0.11	0.02	0.05	0.10	0.14	0.07	0.16	0.08	0.17	0.08
37	JN.1	0.02	0.12	0.05	0.08	0.02	NA	0.13	0.04	0.08	0.07	0.29	0.11	0.11	0.02	0.05	0.10	0.14	0.07	0.16	0.08	0.17	0.08

This supplementary material is hosted by Eurosurveillance as supporting information alongside the article 'Early detection of the emerging BA.2.86 lineage in Sweden through integrated genomic surveillance of wastewater and SARS-CoV-2 cases', on behalf of the authors, who remain responsible for the accuracy and appropriateness of the content. The same standards for ethics, copyright, attributions and permissions as for the article apply. Supplements are not edited by Eurosurveillance and the journal is not responsible for the maintenance of any links or email addresses provided therein.

	Freyja		Relative abundance of BA.2.86-specific markers																				
week	lineage	abundance	T15756A	ins21608	C28958A	Δ23009-23011	A22556G	C897A	A26610G	A7842G	G21941T	C21711T	T22896A	G11042T	C23271T	C22353A	G26529C	C22916T	C24990T	G8393A	G21624C	T22032C	Δ2ss2194-22196
37	BA.2.86.3	0.00	0.12	0.05	0.08	0.02	NA	0.13	0.04	0.08	0.07	0.29	0.11	0.11	0.02	0.05	0.10	0.14	0.07	0.16	0.08	0.17	0.08
36	JN.2	0.05	0.16	NA	0.23	0.01	0.12	0.15	0.10	0.10	0.07	0.27	0.08	0.07	0.05	0.05	0.09	0.11	0.07	0.11	NA	0.18	0.09
36	JN.1	0.02	0.16	NA	0.23	0.01	0.12	0.15	0.10	0.10	0.07	0.27	0.08	0.07	0.05	0.05	0.09	0.11	0.07	0.11	NA	0.18	0.09
36	BA.2.86.3	0.00	0.16	NA	0.23	0.01	0.12	0.15	0.10	0.10	0.07	0.27	0.08	0.07	0.05	0.05	0.09	0.11	0.07	0.11	NA	0.18	0.09
35	JN.2	0.04	0.06	NA	0.11	0.05	NA	0.03	0.24	0.16	0.03	0.07	0.14	0.05	0.08	0.03	0.11	0.18	0.08	0.10	0.06	0.10	0.06
35	JN.1	0.02	0.06	NA	0.11	0.05	NA	0.03	0.24	0.16	0.03	0.07	0.14	0.05	0.08	0.03	0.11	0.18	0.08	0.10	0.06	0.10	0.06
35	BA.2.86.3	0.00	0.06	NA	0.11	0.05	NA	0.03	0.24	0.16	0.03	0.07	0.14	0.05	0.08	0.03	0.11	0.18	0.08	0.10	0.06	0.10	0.06
Stockholm-Käppala																							
38	JN.1	0.04	0.22	NA	0.08	NA	0.05	0.06	0.06	0.07	0.06	0.22	0.13	0.11	0.04	0.12	0.09	0.18	0.09	0.11	0.07	0.19	0.08
38	JN.2	0.02	0.22	NA	0.08	NA	0.05	0.06	0.06	0.07	0.06	0.22	0.13	0.11	0.04	0.12	0.09	0.18	0.09	0.11	0.07	0.19	0.08
38	BA.2.86.3	0.00	0.22	NA	0.08	NA	0.05	0.06	0.06	0.07	0.06	0.22	0.13	0.11	0.04	0.12	0.09	0.18	0.09	0.11	0.07	0.19	0.08
36	JN.2	0.03	0.05	NA	0.08	NA	0.09	0.03	0.06	0.05	0.05	0.14	0.08	0.06	0.02	0.09	0.09	0.12	0.08	0.07	0.05	0.13	0.03
36	JN.1	0.01	0.05	NA	0.08	NA	0.09	0.03	0.06	0.05	0.05	0.14	0.08	0.06	0.02	0.09	0.09	0.12	0.08	0.07	0.05	0.13	0.03
35	JN.1	0.03	0.14	NA	0.06	NA	NA	0.05	0.02	0.07	0.04	0.09	0.07	0.08	NA	0.03	0.15	0.11	0.08	0.13	0.03	0.14	NA
35	JN.2	0.01	0.14	NA	0.06	NA	NA	0.05	0.02	0.07	0.04	0.09	0.07	0.08	NA	0.03	0.15	0.11	0.08	0.13	0.03	0.14	NA
Stockholm-Henriksdal																							
37	JN.2	0.03	NA	NA	0.10	NA	NA	0.07	NA	0.05	0.04	NA	0.11	0.06	NA	0.06	0.07	0.12	0.06	0.10	NA	0.14	NA
37	JN.1	0.01	NA	NA	0.10	NA	NA	0.07	NA	0.05	0.04	NA	0.11	0.06	NA	0.06	0.07	0.12	0.06	0.10	NA	0.14	NA

This supplementary material is hosted by Eurosurveillance as supporting information alongside the article 'Early detection of the emerging BA.2.86 lineage in Sweden through integrated genomic surveillance of wastewater and SARS-CoV-2 cases', on behalf of the authors, who remain responsible for the accuracy and appropriateness of the content. The same standards for ethics, copyright, attributions and permissions as for the article apply. Supplements are not edited by Eurosurveillance and the journal is not responsible for the maintenance of any links or email addresses provided therein.

	Freyja		Relative abundance of BA.2.86-specific markers																				
week	lineage	abundance	T15756A	ins21608	C28958A	Δ23009-23011	A22556G	C897A	A26610G	A7842G	G21941T	C21711T	T22896A	G11042T	C23271T	C22353A	G26529C	C22916T	C24990T	G8393A	G21624C	T22032C	Δ2ss2194-22196
36	JN.2	0.04	0.14	NA	0.08	0.02	0.08	0.06	0.04	0.03	0.09	0.29	0.07	0.04	0.02	0.04	0.06	0.11	0.05	0.11	0.05	0.18	0.07
36	JN.1	0.02	0.14	NA	0.08	0.02	0.08	0.06	0.04	0.03	0.09	0.29	0.07	0.04	0.02	0.04	0.06	0.11	0.05	0.11	0.05	0.18	0.07
35	JN.1	0.03	0.10	NA	0.08	NA	NA	0.04	NA	0.06	0.09	0.11	0.11	0.07	NA	0.04	NA	0.15	0.06	0.08	NA	0.16	NA
Stockholm-Grödinge																							
38	JN.2	0.03	0.22	NA	0.09	0.01	NA	0.09	0.05	0.11	0.06	0.47	0.08	0.07	0.02	0.04	0.05	0.10	0.07	0.12	NA	0.20	0.05
38	JN.1	0.02	0.22	NA	0.09	0.01	NA	0.09	0.05	0.11	0.06	0.47	0.08	0.07	0.02	0.04	0.05	0.10	0.07	0.12	NA	0.20	0.05
37	JN.2	0.05	0.14	NA	0.11	0.01	0.10	0.08	0.06	0.04	0.07	0.14	0.12	0.11	0.06	0.08	0.08	0.15	0.08	0.09	0.05	0.15	0.03
37	JN.1	0.02	0.14	NA	0.11	0.01	0.10	0.08	0.06	0.04	0.07	0.14	0.12	0.11	0.06	0.08	0.08	0.15	0.08	0.09	0.05	0.15	0.03
36	JN.2	0.02	0.16	NA	0.06	NA	0.04	0.05	0.08	0.06	0.05	0.09	0.06	0.04	0.02	0.03	0.08	0.09	0.05	0.08	0.04	0.17	NA
36	JN.1	0.02	0.16	NA	0.06	NA	0.04	0.05	0.08	0.06	0.05	0.09	0.06	0.04	0.02	0.03	0.08	0.09	0.05	0.08	0.04	0.17	NA
Stockholm-Bromma																							
38	JN.1	0.02	0.21	NA	0.07	NA	NA	0.08	NA	0.07	0.03	NA	0.08	0.05	NA	0.06	0.05	0.11	0.04	0.08	NA	0.17	NA
38	JN.2	0.02	0.21	NA	0.07	NA	NA	0.08	NA	0.07	0.03	NA	0.08	0.05	NA	0.06	0.05	0.11	0.04	0.08	NA	0.17	NA
37	JN.2	0.02	NA	0.03	0.06	NA	0.06	0.04	NA	0.04	0.06	0.29	0.10	0.07	0.02	0.04	0.04	0.14	0.08	0.06	0.05	0.15	0.04
37	JN.1	0.02	NA	0.03	0.06	NA	0.06	0.04	NA	0.04	0.06	0.29	0.10	0.07	0.02	0.04	0.04	0.14	0.08	0.06	0.05	0.15	0.04
37	BA.2.86.3	0.00	NA	0.03	0.06	NA	0.06	0.04	NA	0.04	0.06	0.29	0.10	0.07	0.02	0.04	0.04	0.14	0.08	0.06	0.05	0.15	0.04
36	JN.2	0.02	0.12	NA	0.06	NA	0.08	0.05	0.04	0.07	0.06	0.24	0.07	0.04	0.02	0.06	0.06	0.10	0.08	0.11	0.04	0.14	NA
36	JN.1	0.02	0.12	NA	0.06	NA	0.08	0.05	0.04	0.07	0.06	0.24	0.07	0.04	0.02	0.06	0.06	0.10	0.08	0.11	0.04	0.14	NA

This supplementary material is hosted by Eurosurveillance as supporting information alongside the article 'Early detection of the emerging BA.2.86 lineage in Sweden through integrated genomic surveillance of wastewater and SARS-CoV-2 cases', on behalf of the authors, who remain responsible for the accuracy and appropriateness of the content. The same standards for ethics, copyright, attributions and permissions as for the article apply. Supplements are not edited by Eurosurveillance and the journal is not responsible for the maintenance of any links or email addresses provided therein.

	Freyja		Relative abundance of BA.2.86-specific markers																				
week	lineage	abundance	T15756A	ins21608	C28958A	Δ23009-23011	A22556G	C897A	A26610G	A7842G	G21941T	C21711T	T22896A	G11042T	C23271T	C22353A	G26529C	C22916T	C24990T	G8393A	G21624C	T22032C	Δ2ss2194-22196
Malmö																							
38	JN.3	0.03	0.11	NA	0.06	NA	NA	0.05	0.05	0.08	0.03	NA	0.06	0.05	NA	0.06	0.07	0.09	0.07	0.11	NA	0.17	NA
38	JN.1	0.02	0.11	NA	0.06	NA	NA	0.05	0.05	0.08	0.03	NA	0.06	0.05	NA	0.06	0.07	0.09	0.07	0.11	NA	0.17	NA
38	BA.2.86.3	0.00	0.11	NA	0.06	NA	NA	0.05	0.05	0.08	0.03	NA	0.06	0.05	NA	0.06	0.07	0.09	0.07	0.11	NA	0.17	NA
37	JN.2	0.05	0.13	NA	0.14	NA	0.09	0.05	0.07	0.05	0.07	0.26	0.12	0.13	0.03	0.08	0.12	0.14	0.07	0.14	0.03	0.17	0.04
37	JN.1	0.02	0.13	NA	0.14	NA	0.09	0.05	0.07	0.05	0.07	0.26	0.12	0.13	0.03	0.08	0.12	0.14	0.07	0.14	0.03	0.17	0.04
37	BA.2.86.3	0.00	0.13	NA	0.14	NA	0.09	0.05	0.07	0.05	0.07	0.26	0.12	0.13	0.03	0.08	0.12	0.14	0.07	0.14	0.03	0.17	0.04
36	NA	NA	0.11	NA	0.06	NA	NA	0.05	0.05	0.08	0.03	NA	0.06	0.05	NA	0.06	0.07	0.09	0.07	0.11	NA	0.17	NA
35	JN.1	0.03	0.12	NA	0.04	NA	NA	0.05	NA	0.04	0.05	0.08	0.08	0.04	NA	0.03	0.06	0.12	0.07	0.08	0.04	0.14	NA
35	BA.2.86.3	0.00	0.12	NA	0.04	NA	NA	0.05	NA	0.04	0.05	0.08	0.08	0.04	NA	0.03	0.06	0.12	0.07	0.08	0.04	0.14	NA
Luleå																							
38	JN.1	0.02	0.18	NA	0.04	NA	NA	0.05	NA	0.06	0.05	0.29	0.07	0.06	0.02	0.07	0.05	0.12	0.07	0.06	NA	0.17	NA
38	JN.2	0.01	0.18	NA	0.04	NA	NA	0.05	NA	0.06	0.05	0.29	0.07	0.06	0.02	0.07	0.05	0.12	0.07	0.06	NA	0.17	NA
38	BA.2.86.3	0.01	0.18	NA	0.04	NA	NA	0.05	NA	0.06	0.05	0.29	0.07	0.06	0.02	0.07	0.05	0.12	0.07	0.06	NA	0.17	NA
37	JN.2	0.08	0.15	NA	0.10	0.02	0.22	0.10	0.15	0.09	0.07	0.28	0.17	0.12	0.07	0.07	0.24	0.22	0.12	0.15	0.05	0.23	NA
37	JN.1	0.02	0.15	NA	0.10	0.02	0.22	0.10	0.15	0.09	0.07	0.28	0.17	0.12	0.07	0.07	0.24	0.22	0.12	0.15	0.05	0.23	NA
37	BA.2.86.3	0.00	0.15	NA	0.10	0.02	0.22	0.10	0.15	0.09	0.07	0.28	0.17	0.12	0.07	0.07	0.24	0.22	0.12	0.15	0.05	0.23	NA
36	JN.1	0.02	0.15	NA	0.10	NA	0.12	0.04	0.05	0.09	0.03	NA	0.05	0.03	NA	0.07	0.07	0.08	0.06	0.18	NA	0.12	NA

This supplementary material is hosted by Eurosurveillance as supporting information alongside the article 'Early detection of the emerging BA.2.86 lineage in Sweden through integrated genomic surveillance of wastewater and SARS-CoV-2 cases', on behalf of the authors, who remain responsible for the accuracy and appropriateness of the content. The same standards for ethics, copyright, attributions and permissions as for the article apply. Supplements are not edited by Eurosurveillance and the journal is not responsible for the maintenance of any links or email addresses provided therein.

	Freyja		Relative abundance of BA.2.86-specific markers																				
week	lineage	abundance	T15756A	ins21608	C28958A	Δ23009-23011	A22556G	C897A	A26610G	A7842G	G21941T	C21711T	T22896A	G11042T	C23271T	C22353A	G26529C	C22916T	C24990T	G8393A	G21624C	T22032C	Δ2ss2194-22196
36	JN.2	0.00	0.15	NA	0.10	NA	0.12	0.04	0.05	0.09	0.03	NA	0.05	0.03	NA	0.07	0.07	0.08	0.06	0.18	NA	0.12	NA
36	BA.2.86.3	0.00	0.15	NA	0.10	NA	0.12	0.04	0.05	0.09	0.03	NA	0.05	0.03	NA	0.07	0.07	0.08	0.06	0.18	NA	0.12	NA
35	JN.2	0.02	0.15	NA	0.09	NA	0.14	0.07	0.07	0.21	0.03	0.53	0.14	0.14	NA	0.04	0.19	0.18	0.05	0.08	NA	0.18	NA
35	JN.1	0.02	0.15	NA	0.09	NA	0.14	0.07	0.07	0.21	0.03	0.53	0.14	0.14	NA	0.04	0.19	0.18	0.05	0.08	NA	0.18	NA
35	BA.2.86.3	0.00	0.15	NA	0.09	NA	0.14	0.07	0.07	0.21	0.03	0.53	0.14	0.14	NA	0.04	0.19	0.18	0.05	0.08	NA	0.18	NA
35	BA.2.86.2	0.00	0.15	NA	0.09	NA	0.14	0.07	0.07	0.21	0.03	0.53	0.14	0.14	NA	0.04	0.19	0.18	0.05	0.08	NA	0.18	NA
Linköping																							
38	JN.2	0.11	0.27	0.06	0.11	0.05	0.24	0.10	0.13	0.17	0.13	0.25	0.14	0.19	0.20	0.14	0.09	0.19	0.10	0.19	0.06	0.19	0.16
38	JN.1	0.02	0.27	0.06	0.11	0.05	0.24	0.10	0.13	0.17	0.13	0.25	0.14	0.19	0.20	0.14	0.09	0.19	0.10	0.19	0.06	0.19	0.16
38	BA.2.86.3	0.01	0.27	0.06	0.11	0.05	0.24	0.10	0.13	0.17	0.13	0.25	0.14	0.19	0.20	0.14	0.09	0.19	0.10	0.19	0.06	0.19	0.16
37	JN.1	0.02	0.13	NA	0.03	NA	NA	0.03	NA	0.05	0.05	0.10	0.09	0.07	NA	0.05	0.05	0.12	0.05	0.06	NA	0.16	NA
37	JN.2	0.01	0.13	NA	0.03	NA	NA	0.03	NA	0.05	0.05	0.10	0.09	0.07	NA	0.05	0.05	0.12	0.05	0.06	NA	0.16	NA
37	BA.2.86.3	0.00	0.13	NA	0.03	NA	NA	0.03	NA	0.05	0.05	0.10	0.09	0.07	NA	0.05	0.05	0.12	0.05	0.06	NA	0.16	NA
36	JN.1	0.02	0.08	NA	0.05	NA	NA	0.04	NA	0.06	0.03	NA	0.04	0.04	NA	0.04	0.06	0.07	0.08	0.11	NA	0.16	NA
36	JN.2	0.01	0.08	NA	0.05	NA	NA	0.04	NA	0.06	0.03	NA	0.04	0.04	NA	0.04	0.06	0.07	0.08	0.11	NA	0.16	NA
36	BA.2.86.3	0.00	0.08	NA	0.05	NA	NA	0.04	NA	0.06	0.03	NA	0.04	0.04	NA	0.04	0.06	0.07	0.08	0.11	NA	0.16	NA
35	JN.1	0.02	0.14	NA	0.07	NA	NA	0.08	NA	0.05	0.05	0.19	0.10	0.05	0.01	0.03	0.03	0.14	0.07	0.07	0.07	0.15	0.03
35	BA.2.86.3	0.00	0.14	NA	0.07	NA	NA	0.08	NA	0.05	0.05	0.19	0.10	0.05	0.01	0.03	0.03	0.14	0.07	0.07	0.07	0.15	0.03

This supplementary material is hosted by Eurosurveillance as supporting information alongside the article 'Early detection of the emerging BA.2.86 lineage in Sweden through integrated genomic surveillance of wastewater and SARS-CoV-2 cases', on behalf of the authors, who remain responsible for the accuracy and appropriateness of the content. The same standards for ethics, copyright, attributions and permissions as for the article apply. Supplements are not edited by Eurosurveillance and the journal is not responsible for the maintenance of any links or email addresses provided therein.

	Freyja		Relative abundance of BA.2.86-specific markers																				
week	lineage	abundance	T15756A	ins21608	C28958A	Δ23009-23011	A22556G	C897A	A26610G	A7842G	G21941T	C21711T	T22896A	G11042T	C23271T	C22353A	G26529C	C22916T	C24990T	G8393A	G21624C	T22032C	Δ2ss2194-22196
35	JN.2	0.00	0.14	NA	0.07	NA	NA	0.08	NA	0.05	0.05	0.19	0.10	0.05	0.01	0.03	0.03	0.14	0.07	0.07	0.07	0.15	0.03
Karlstad																							
38	JN.2	0.05	0.16	NA	0.08	0.02	0.22	0.10	0.05	0.09	0.09	0.48	0.10	0.13	0.12	0.09	0.05	0.14	0.07	0.10	0.04	0.21	0.04
38	JN.1	0.02	0.16	NA	0.08	0.02	0.22	0.10	0.05	0.09	0.09	0.48	0.10	0.13	0.12	0.09	0.05	0.14	0.07	0.10	0.04	0.21	0.04
38	BA.2.86.3	0.00	0.16	NA	0.08	0.02	0.22	0.10	0.05	0.09	0.09	0.48	0.10	0.13	0.12	0.09	0.05	0.14	0.07	0.10	0.04	0.21	0.04
37	JN.2	0.08	0.11	NA	0.11	0.03	0.22	0.08	0.06	0.09	0.10	0.28	0.13	0.13	0.07	0.08	0.08	0.17	0.09	0.14	0.05	0.15	0.09
37	JN.1	0.02	0.11	NA	0.11	0.03	0.22	0.08	0.06	0.09	0.10	0.28	0.13	0.13	0.07	0.08	0.08	0.17	0.09	0.14	0.05	0.15	0.09
36	JN.2	0.08	0.13	NA	0.11	0.04	0.17	0.09	0.05	0.10	0.09	0.20	0.11	0.11	0.11	0.09	0.09	0.17	0.08	0.19	0.03	0.17	0.11
36	JN.1	0.02	0.13	NA	0.11	0.04	0.17	0.09	0.05	0.10	0.09	0.20	0.11	0.11	0.11	0.09	0.09	0.17	0.08	0.19	0.03	0.17	0.11
35	JN.1	0.03	0.07	NA	0.08	0.02	0.07	0.08	NA	0.05	0.05	0.10	0.09	0.10	0.05	0.05	0.06	0.12	0.09	0.09	0.06	0.10	NA
35	JN.2	0.02	0.07	NA	0.08	0.02	0.07	0.08	NA	0.05	0.05	0.10	0.09	0.10	0.05	0.05	0.06	0.12	0.09	0.09	0.06	0.10	NA
Kalmar																							
38	JN.2	0.03	0.14	NA	0.07	0.02	NA	0.05	0.09	0.05	0.05	NA	0.09	0.09	NA	0.06	0.06	0.13	0.05	0.08	NA	0.16	NA
38	JN.1	0.01	0.14	NA	0.07	0.02	NA	0.05	0.09	0.05	0.05	NA	0.09	0.09	NA	0.06	0.06	0.13	0.05	0.08	NA	0.16	NA
38	BA.2.86.3	0.00	0.14	NA	0.07	0.02	NA	0.05	0.09	0.05	0.05	NA	0.09	0.09	NA	0.06	0.06	0.13	0.05	0.08	NA	0.16	NA
36	JN.1	0.02	0.09	NA	0.05	NA	NA	0.03	NA	0.04	0.03	0.24	0.05	0.03	NA	0.04	0.05	0.07	0.05	0.08	0.05	0.13	NA
36	JN.2	0.01	0.09	NA	0.05	NA	NA	0.03	NA	0.04	0.03	0.24	0.05	0.03	NA	0.04	0.05	0.07	0.05	0.08	0.05	0.13	NA
36	BA.2.86.3	0.00	0.09	NA	0.05	NA	NA	0.03	NA	0.04	0.03	0.24	0.05	0.03	NA	0.04	0.05	0.07	0.05	0.08	0.05	0.13	NA

This supplementary material is hosted by Eurosurveillance as supporting information alongside the article 'Early detection of the emerging BA.2.86 lineage in Sweden through integrated genomic surveillance of wastewater and SARS-CoV-2 cases', on behalf of the authors, who remain responsible for the accuracy and appropriateness of the content. The same standards for ethics, copyright, attributions and permissions as for the article apply. Supplements are not edited by Eurosurveillance and the journal is not responsible for the maintenance of any links or email addresses provided therein.

	Freyja		Relative abundance of BA.2.86-specific markers																				
week	lineage	abundance	T15756A	ins21608	C28958A	Δ23009-23011	A22556G	C897A	A26610G	A7842G	G21941T	C21711T	T22896A	G11042T	C23271T	C22353A	G26529C	C22916T	C24990T	G8393A	G21624C	T22032C	Δ2ss2194-22196
35	JN.1	0.02	0.11	NA	0.06	NA	NA	0.05	NA	0.05	0.04	NA	0.10	0.08	NA	0.05	0.04	0.14	0.08	0.08	0.05	0.12	NA
35	BA.2.86.2	0.02	0.11	NA	0.06	NA	NA	0.05	NA	0.05	0.04	NA	0.10	0.08	NA	0.05	0.04	0.14	0.08	0.08	0.05	0.12	NA
35	BA.2.86.3	0.00	0.11	NA	0.06	NA	NA	0.05	NA	0.05	0.04	NA	0.10	0.08	NA	0.05	0.04	0.14	0.08	0.08	0.05	0.12	NA
Jönköping																							
37	JN.1	0.01	NA	NA	NA	NA	NA	NA	NA	NA	0.06	NA	0.10	0.09	NA	NA	NA	0.14	NA	0.06	NA	0.11	NA
37	JN.2	0.00	NA	NA	NA	NA	NA	NA	NA	NA	0.06	NA	0.10	0.09	NA	NA	NA	0.14	NA	0.06	NA	0.11	NA
36	BA.2.86.2	0.01	0.14	NA	NA	NA	NA	0.04	NA	0.03	0.04	NA	0.08	NA	NA	NA	0.05	0.11	0.06	0.05	NA	0.17	NA
36	JN.1	0.01	0.14	NA	NA	NA	NA	0.04	NA	0.03	0.04	NA	0.08	NA	NA	NA	0.05	0.11	0.06	0.05	NA	0.17	NA
36	JN.2	0.00	0.14	NA	NA	NA	NA	0.04	NA	0.03	0.04	NA	0.08	NA	NA	NA	0.05	0.11	0.06	0.05	NA	0.17	NA
36	BA.2.86.3	0.00	0.14	NA	NA	NA	NA	0.04	NA	0.03	0.04	NA	0.08	NA	NA	NA	0.05	0.11	0.06	0.05	NA	0.17	NA
35	JN.1	0.01	NA	NA	NA	NA	NA	0.07	NA	NA	NA	NA	0.07	NA	NA	NA	NA	0.10	0.06	0.08	NA	0.20	NA
35	BA.2.86.2	0.01	NA	NA	NA	NA	NA	0.07	NA	NA	NA	NA	0.07	NA	NA	NA	NA	0.10	0.06	0.08	NA	0.20	NA
Helsingborg																							
38	JN.2	0.02	0.15	NA	0.04	NA	NA	0.06	NA	0.08	0.03	NA	0.09	0.08	NA	0.06	0.04	0.13	0.07	0.08	NA	0.14	NA
38	JN.1	0.02	0.15	NA	0.04	NA	NA	0.06	NA	0.08	0.03	NA	0.09	0.08	NA	0.06	0.04	0.13	0.07	0.08	NA	0.14	NA
37	BA.2.86.3	0.02	0.08	NA	0.07	NA	NA	0.05	NA	0.05	0.04	NA	0.10	0.08	NA	0.06	0.07	0.14	0.08	0.08	NA	0.12	NA
37	JN.1	0.02	0.08	NA	0.07	NA	NA	0.05	NA	0.05	0.04	NA	0.10	0.08	NA	0.06	0.07	0.14	0.08	0.08	NA	0.12	NA
36	JN.1	0.02	0.14	NA	0.09	NA	NA	0.02	NA	0.05	0.02	NA	0.06	0.03	NA	0.04	0.09	0.08	0.05	0.11	NA	0.13	NA

This supplementary material is hosted by Eurosurveillance as supporting information alongside the article 'Early detection of the emerging BA.2.86 lineage in Sweden through integrated genomic surveillance of wastewater and SARS-CoV-2 cases', on behalf of the authors, who remain responsible for the accuracy and appropriateness of the content. The same standards for ethics, copyright, attributions and permissions as for the article apply. Supplements are not edited by Eurosurveillance and the journal is not responsible for the maintenance of any links or email addresses provided therein.

	Freyja		Relative abundance of BA.2.86-specific markers																				
week	lineage	abundance	T15756A	ins21608	C28958A	Δ23009-23011	A22556G	C897A	A26610G	A7842G	G21941T	C21711T	T22896A	G11042T	C23271T	C22353A	G26529C	C22916T	C24990T	G8393A	G21624C	T22032C	Δ2ss2194-22196
36	BA.2.86.3	0.01	0.14	NA	0.09	NA	NA	0.02	NA	0.05	0.02	NA	0.06	0.03	NA	0.04	0.09	0.08	0.05	0.11	NA	0.13	NA
36	JN.3	0.00	0.14	NA	0.09	NA	NA	0.02	NA	0.05	0.02	NA	0.06	0.03	NA	0.04	0.09	0.08	0.05	0.11	NA	0.13	NA
35	JN.1	0.02	0.19	NA	0.04	0.01	0.05	0.11	0.04	0.07	0.08	NA	0.09	0.11	0.04	0.05	0.06	0.13	0.08	0.12	NA	0.10	NA
35	JN.2	0.02	0.19	NA	0.04	0.01	0.05	0.11	0.04	0.07	0.08	NA	0.09	0.11	0.04	0.05	0.06	0.13	0.08	0.12	NA	0.10	NA
35	BA.2.86.3	0.00	0.19	NA	0.04	0.01	0.05	0.11	0.04	0.07	0.08	NA	0.09	0.11	0.04	0.05	0.06	0.13	0.08	0.12	NA	0.10	NA
Göteborg																							
38	JN.3	0.02	0.24	NA	0.06	NA	NA	0.05	NA	0.04	0.03	NA	0.07	0.06	NA	0.05	0.05	0.09	0.08	0.05	NA	0.15	NA
38	JN.1	0.02	0.24	NA	0.06	NA	NA	0.05	NA	0.04	0.03	NA	0.07	0.06	NA	0.05	0.05	0.09	0.08	0.05	NA	0.15	NA
38	BA.2.86.3	0.00	0.24	NA	0.06	NA	NA	0.05	NA	0.04	0.03	NA	0.07	0.06	NA	0.05	0.05	0.09	0.08	0.05	NA	0.15	NA
37	JN.1	0.02	0.10	NA	0.05	NA	0.07	0.04	NA	0.04	0.04	0.25	0.09	0.08	0.03	0.07	0.05	0.12	0.06	0.08	NA	0.16	0.03
37	JN.2	0.02	0.10	NA	0.05	NA	0.07	0.04	NA	0.04	0.04	0.25	0.09	0.08	0.03	0.07	0.05	0.12	0.06	0.08	NA	0.16	0.03
37	BA.2.86.3	0.00	0.10	NA	0.05	NA	0.07	0.04	NA	0.04	0.04	0.25	0.09	0.08	0.03	0.07	0.05	0.12	0.06	0.08	NA	0.16	0.03
36	JN.1	0.02	0.19	NA	0.07	NA	NA	0.03	NA	0.04	0.03	0.19	0.04	0.02	0.02	0.03	0.06	0.07	0.03	0.06	0.03	0.16	0.03
36	JN.2	0.01	0.19	NA	0.07	NA	NA	0.03	NA	0.04	0.03	0.19	0.04	0.02	0.02	0.03	0.06	0.07	0.03	0.06	0.03	0.16	0.03
36	BA.2.86.3	0.00	0.19	NA	0.07	NA	NA	0.03	NA	0.04	0.03	0.19	0.04	0.02	0.02	0.03	0.06	0.07	0.03	0.06	0.03	0.16	0.03
35	JN.1	0.03	0.12	NA	0.05	NA	0.04	0.06	NA	0.06	0.04	0.05	0.08	0.05	NA	0.04	0.08	0.10	0.07	0.10	NA	0.14	NA
35	JN.2	0.01	0.12	NA	0.05	NA	0.04	0.06	NA	0.06	0.04	0.05	0.08	0.05	NA	0.04	0.08	0.10	0.07	0.10	NA	0.14	NA
35	BA.2.86.3	0.00	0.12	NA	0.05	NA	0.04	0.06	NA	0.06	0.04	0.05	0.08	0.05	NA	0.04	0.08	0.10	0.07	0.10	NA	0.14	NA

This supplementary material is hosted by Eurosurveillance as supporting information alongside the article 'Early detection of the emerging BA.2.86 lineage in Sweden through integrated genomic surveillance of wastewater and SARS-CoV-2 cases', on behalf of the authors, who remain responsible for the accuracy and appropriateness of the content. The same standards for ethics, copyright, attributions and permissions as for the article apply. Supplements are not edited by Eurosurveillance and the journal is not responsible for the maintenance of any links or email addresses provided therein.

	Freyja		Relative abundance of BA.2.86-specific markers																				
week	lineage	abundance	T15756A	ins21608	C28958A	Δ23009-23011	A22556G	C897A	A26610G	A7842G	G21941T	C21711T	T22896A	G11042T	C23271T	C22353A	G26529C	C22916T	C24990T	G8393A	G21624C	T22032C	Δ2ss2194-22196
Gävle																							
38	JN.2	0.02	0.16	NA	0.06	NA	0.04	0.07	NA	0.07	0.04	0.26	0.09	0.06	0.02	0.07	0.07	0.11	0.09	0.06	0.05	0.17	NA
38	JN.1	0.02	0.16	NA	0.06	NA	0.04	0.07	NA	0.07	0.04	0.26	0.09	0.06	0.02	0.07	0.07	0.11	0.09	0.06	0.05	0.17	NA
37	JN.2	0.02	0.10	NA	0.05	0.01	NA	0.07	NA	0.06	0.05	NA	0.10	0.09	NA	0.04	0.05	0.12	0.07	0.08	NA	0.13	NA
37	JN.1	0.02	0.10	NA	0.05	0.01	NA	0.07	NA	0.06	0.05	NA	0.10	0.09	NA	0.04	0.05	0.12	0.07	0.08	NA	0.13	NA
37	BA.2.86.3	0.00	0.10	NA	0.05	0.01	NA	0.07	NA	0.06	0.05	NA	0.10	0.09	NA	0.04	0.05	0.12	0.07	0.08	NA	0.13	NA
36	JN.1	0.03	NA	NA	0.04	NA	0.05	0.05	NA	0.06	0.06	NA	0.11	0.09	0.03	0.05	0.06	0.17	0.07	0.09	0.11	0.14	NA
36	BA.2.86.3	0.01	NA	NA	0.04	NA	0.05	0.05	NA	0.06	0.06	NA	0.11	0.09	0.03	0.05	0.06	0.17	0.07	0.09	0.11	0.14	NA
36	JN.2	0.01	NA	NA	0.04	NA	0.05	0.05	NA	0.06	0.06	NA	0.11	0.09	0.03	0.05	0.06	0.17	0.07	0.09	0.11	0.14	NA

This supplementary material is hosted by Eurosurveillance as supporting information alongside the article 'Early detection of the emerging BA.2.86 lineage in Sweden through integrated genomic surveillance of wastewater and SARS-CoV-2 cases', on behalf of the authors, who remain responsible for the accuracy and appropriateness of the content. The same standards for ethics, copyright, attributions and permissions as for the article apply. Supplements are not edited by Eurosurveillance and the journal is not responsible for the maintenance of any links or email addresses provided therein.

Supplementary Table S1. Description of BA.2.86 cases during weeks 31-38, 2023, Sweden.

	**BA.2.86**		**Other lineages**		**P-value**
Indicator	n	%	n	%	
Total[a]	70		2171		-
Median age in years (range)	80 (0-100+)		77 (0-100+)		0.27[b]
Female	36	51	1080	50	0.78[c]
Hospitalisation in ward	21	30	617	28	0.77c
Hospitalisation ICU	1	1.4	31	1.4	1.0[d]
Death within 30 days	5	7.1	118	5.4	0.59[d]
Median time since most recent vaccination in days (range)	285 (97-813)		323 (16-911)		0.90[b]
Last dose less than 180 days before infection	45	64	1367	63	0.82[b]
Unvaccinated	6	8.6	255	11.8	0.57[d]

a Number of cases during the study period that were assigned a lineages and where registry data was available (86 % of all cases assigned a lineages during the study period)

b Kruskall-Wallis test

c Chi-squared test

d Fischers exact test

This supplementary material is hosted by Eurosurveillance as supporting information alongside the article 'Early detection of the emerging BA.2.86 lineage in Sweden through integrated genomic surveillance of wastewater and SARS-CoV-2 cases', on behalf of the authors, who remain responsible for the accuracy and appropriateness of the content. The same standards for ethics, copyright, attributions and permissions as for the article apply. Supplements are not edited by Eurosurveillance and the journal is not responsible for the maintenance of any links or email addresses provided therein.

Supplementary materials

Supplementary Material. GISAID's EpiCoV database accession identifiers for all Swedish BA.2.86 cases

EPI_ISL_18147545, EPI_ISL_18151536, EPI_ISL_18248380, EPI_ISL_18248383, EPI_ISL_18147561, EPI_ISL_18168405, EPI_ISL_18147559, EPI_ISL_18221983, EPI_ISL_18221980, EPI_ISL_18221984, EPI_ISL_18258538, EPI_ISL_18259068, EPI_ISL_18259100, EPI_ISL_18259115, EPI_ISL_18259108, EPI_ISL_18281975, EPI_ISL_18258923, EPI_ISL_18338939, EPI_ISL_18367677, EPI_ISL_18249308, EPI_ISL_18248384, EPI_ISL_18281969, EPI_ISL_18258926, EPI_ISL_18258950, EPI_ISL_18281993, EPI_ISL_18286753, EPI_ISL_18274638, EPI_ISL_18338924, EPI_ISL_18338911, EPI_ISL_18295098, EPI_ISL_18308751, EPI_ISL_18352308, EPI_ISL_18295117, EPI_ISL_18338928, EPI_ISL_18367697, EPI_ISL_18367698, EPI_ISL_18308760, EPI_ISL_18308767, EPI_ISL_18338787, EPI_ISL_18338882, EPI_ISL_18338901, EPI_ISL_18338935, EPI_ISL_18316107, EPI_ISL_18338875, EPI_ISL_18338938, EPI_ISL_18338707, EPI_ISL_18316068, EPI_ISL_18338805, EPI_ISL_18338748, EPI_ISL_18338766, EPI_ISL_18338783, EPI_ISL_18338706, EPI_ISL_18303513, EPI_ISL_18338793, EPI_ISL_18338932, EPI_ISL_18316061, EPI_ISL_18338879, EPI_ISL_18338881, EPI_ISL_18338944, EPI_ISL_18338907, EPI_ISL_18338942, EPI_ISL_18332902, EPI_ISL_18331288, EPI_ISL_18331289, EPI_ISL_18332979, EPI_ISL_18331299, EPI_ISL_18331300, EPI_ISL_18359973, EPI_ISL_18399641, EPI_ISL_18367414 ,EPI_ISL_18367416

Supplementary methods

BA.2.86 marker specificity investigation using the GISAID-EpiCoV database

A list of 50 BA.2.86 branch-specific markers was formulated from:

- **i)** GitHub cov-lineages / pango-designation issue #2183 [1], providing 40 nucleotide substitutions, four deletions and one insertion.
- **ii)** UShER / Cov2Tree, providing 47 nucleotide substitutions [2], of which 37 were in common with the GitHub issue above.

The total number of hits present in the GISAID dataset was obtained for each marker from the entire dataset. Metadata of each accession number containing at least one of the mutations was extracted from 1 July 2023 using the GISAIDR package in R [3]. When the number of GISAID hits since 1 July 2023 exceeded 2000 for a specific marker, only metadata from the most recent 2000 hits was extracted. Five markers reverted to the original reference (A12160G, C23013A, A23604G, A26529C, T9866C) were excluded from the analysis due to incompatibility with the GISAIDR search and download options. The percentage of GISAID hits for each marker across SARS-CoV-2 variants is shown in Figure S1.

Wastewater sample collection and processing

For all places, untreated wastewater integrated samples representative of a single day (24 hours) were collected weekly by flow compensated samplers. All measurements represent only one day except for Uppsala, where the measurements represent one week, as samples

This supplementary material is hosted by Eurosurveillance as supporting information alongside the article 'Early detection of the emerging BA.2.86 lineage in Sweden through integrated genomic surveillance of wastewater and SARS-CoV-2 cases', on behalf of the authors, who remain responsible for the accuracy and appropriateness of the content. The same standards for ethics, copyright, attributions and permissions as for the article apply. Supplements are not edited by Eurosurveillance and the journal is not responsible for the maintenance of any links or email addresses provided therein.

were collected each day and then combined flow-proportionally into one composite weekly sample. The samples were processed according to the methods described in Isaksson et al. (2022) [4]. Briefly, the viral genomic material was concentrated and extracted by the direct capture method using the Maxwell RSC Enviro TNA kit (Promega). Absolute quantification of the copy numbers of the SARS-CoV-2 genome is performed by One-Step RT-qPCR using the Flu SC2 Multiplex Assay (CDC). To correct for variations in population size and wastewater flow, the SARS-CoV-2 genome copy numbers are normalized by pepper mild mottle virus (PMMoV) copy numbers. PMMoV is quantified using a modified version of the assay of Zhang et al. (2006) [5].

Virus genome sequencing of wastewater samples

Wastewater samples collected and processed according to Isaksson et al. (2022) [4] were sequenced either on Illumina Miseq platform (weeks 22-34), or Thermo Fisher's Ion S5 XL System (weeks 35-38).

Miseq Sequencing

DNAse treatment (DNase I, Amplification Grade, Invitrogen) was performed to reduce unspecific amplification before library preparation for Miseq sequencing sequencing for samples between week 30-34 . The COVIDSeq Assay kit (Illumina) was used to generate the libraries. Samples from weeks 22, 23, 33, and 34 were amplified using the Artic V4.1 NCOV-2019 Panel primers (IDTDNA), while the remaining weeks were amplified using the Artic V5.3.2 NCOV-2019 Panel primers (IDTDNA). The resulting libraries were sequenced on the Illumina Miseq platform using the MiSeq Reagent Kit v3 2 x 76 (150-cycle) (Illumina) at the Functional Microbial Ecology Lab (FUME) within the Swedish University of Agriculture.

Ion torrent sequencing

Libraries of the extracted total nucleic acids were prepared using the Ion AmpliSeq™ SARS-COV-2 Insight Research Assay (Thermo Fisher Scientific) and the SuperScript™ VILO™ cDNA Synthesis Kit (Thermo Fisher), following the manufacturer's protocol Reverse transcribe RNA with the SuperScript™ VILO™ cDNA Synthesis Kit" (Thermo Fisher) and "Prepare libraries on the Ion Chef™ Instrument" (Thermo Fisher) using the Ion Chef System. Sequencing was performed on the Ion S5 XL System (Thermo Fisher) multiplexing using Torrent Suite Software version 5.16.1 configured according to the manufacturer's protocol (Thermo Fisher). Both library preparation and Ion Torrent sequencingwere performed at the National Genomics Infrastructure (NGI) at Science for Life Laboratory, Uppsala, Sweden.

Wastewater sequence data analysis, identification of mutations using iVar and assignment of variants using Freyja

Briefly, sequencing reads were aligned using bwa mem 0.7.17-r1188 [6] to the reference genome [7], trimmed with iVar 1.4.2 [8] and sorted with samtools 1.17 [9]. Variant calling was performed with iVar using depth files generated with samtools mpileup. The output was then used with Freyja 1.4.7 [10], barcode version 10_18_2023-00-48, to retrieve relative lineage abundances. Freyja relative lineage abundances were included for samples which had more than 40% of the sites with 10x coverage.

Whole genome sequencing of SARS-CoV-2 cases in Sweden

Sweden has a decentralised healthcare system managed independently by its 21 regions. The Public Health Agency of Sweden collects and analyses sequencing data from clinical microbiological laboratories across the country. The sequencing technology utilized at each microbiology laboratory in Sweden is stated in the GISAID accession number of every sequence referenced in this study.

Visualisation

Figures 1 and 2 were generated in R v.4.3.0 using the tidyverse package [11]. Figure 3 was generated adapting maps available at Statistics Sweden with Adobe Illustrator [12, 13].

This supplementary material is hosted by Eurosurveillance as supporting information alongside the article 'Early detection of the emerging BA.2.86 lineage in Sweden through integrated genomic surveillance of wastewater and SARS-CoV-2 cases', on behalf of the authors, who remain responsible for the accuracy and appropriateness of the content. The same standards for ethics, copyright, attributions and permissions as for the article apply. Supplements are not edited by Eurosurveillance and the journal is not responsible for the maintenance of any links or email addresses provided therein.

References

1. GitHub. Cov-lineages / pango-designation. Issue #2183: 2nd-Generation BA.2 saltation lineage, >30 spike mutations (3 seq, 2 countries, Aug 14). 2023. Available from: https://github.com/cov-lineages/pango-designation/issues/2183
2. McBroome J, Thornlow B, Hinrichs AS, Kramer A, De Maio N, Goldman N, et al. A Daily-Updated Database and Tools for Comprehensive SARS-CoV-2 Mutation-Annotated Trees. Mol Biol Evol. 2021;38(12):5819-24. https://doi.org/10.1093/molbev/msab264
3. Wirth W, Duchene S. GISAIDR: Programmatically interact with the GISAID databases. Zenodo. 2022. https://doi.org/10.5281/zenodo.6474693
4. Isaksson F, Lundy L, Hedström A, Székely AJ, Mohamed N. Evaluating the Use of Alternative Normalization Approaches on SARS-CoV-2 Concentrations in Wastewater: Experiences from Two Catchments in Northern Sweden. Environments. 2022;9(3):39. https://doi.org/10.3390/environments9030039
5. Zhang T, Breitbart M, Lee WH, Run JQ, Wei CL, Soh SW, et al. RNA viral community in human feces: prevalence of plant pathogenic viruses. PLoS Biol. 2006;4(1):e3. https://doi.org/10.1371/journal.pbio.0040003
6. Li H, Durbin R. Fast and accurate short read alignment with Burrows-Wheeler transform. Bioinformatics. 2009;25(14):1754-60. https://doi.org/10.1093/bioinformatics/btp324
7. Wu F, Zhao S, Yu B, Chen Y-M, Wang W, Song Z-G, et al. A new coronavirus associated with human respiratory disease in China. Nature. 2020;579(7798):265-9. https://doi.org/10.1038/s41586-020-2008-3
8. Grubaugh ND, Gangavarapu K, Quick J, Matteson NL, De Jesus JG, Main BJ, et al. An amplicon-based sequencing framework for accurately measuring intrahost virus diversity using PrimalSeq and iVar. Genome Biology. 2019;20(1):8. https://doi.org/10.1186/s13059-018-1618-7
9. Li H, Handsaker B, Wysoker A, Fennell T, Ruan J, Homer N, et al. The Sequence Alignment/Map format and SAMtools. Bioinformatics. 2009;25(16):2078-9. https://doi.org/10.1093/bioinformatics/btp352
10. Karthikeyan S, Levy JI, De Hoff P, Humphrey G, Birmingham A, Jepsen K, et al. Wastewater sequencing reveals early cryptic SARS-CoV-2 variant transmission. Nature. 2022;609(7925):101-8. https://doi.org/10.1038/s41586-022-05049-6
11. Wickham H, Averick M, Bryan J, Chang W, McGowan LDA, François R, et al. Welcome to the Tidyverse. Journal of open source software. 2019;4(43):1686. https://doi.org/10.21105/joss.01686
12. Adobe Inc. Adobe Illustrator. 2019. https://adobe.com/products/illustrator
13. Statistics Sweden (Statistikmyndigheten). 2023. https://www.scb.se/
